# Regeneration of the Eyespot and Flagellum in *Euglena gracilis* during Cell Division

**DOI:** 10.3390/plants10102004

**Published:** 2021-09-24

**Authors:** Kazunari Ozasa, Hyunwoong Kang, Simon Song, Shun Tamaki, Tomoko Shinomura, Mizuo Maeda

**Affiliations:** 1 Bioengineering Laboratory, Cluster for Pioneering Research, RIKEN, 2-1 Hirosawa, Wako 351-0198, Saitama, Japan; mizuo@riken.jp; 2 Advanced Laser Processing Research Team, RIKEN Center for Advanced Photonics, RIKEN, 2-1 Hirosawa, Wako 351-0198, Saitama, Japan; 3 Department of Mechanical Engineering, Hanyang University, 222 Wangsimni-ro, Seongdong-gu, Seoul 04763, Korea; kang1026@hanyang.ac.kr (H.K.); simonsong@hanyang.ac.kr (S.S.); 4 Institute of Nano Science and Technology, Hanyang University, 222 Wangsimni-ro, Seongdong-gu, Seoul 04763, Korea; 5 Plant Molecular and Cellular Biology Laboratory, Department of Biosciences, School of Science and Engineering, Teikyo University, 1-1 Toyosatodai, Utsunomiya 320-8551, Tochigi, Japan; tamaki.shun61@gmail.com (S.T.); shinomura@nasu.bio.teikyo-u.ac.jp (T.S.); 6 Microalgae Production Control Technology Laboratory, RIKEN Baton Zone Program, RIKEN, 1-7-22 Suehiro-cho, Tsurumi-ku, Yokohama 230-0045, Kanagawa, Japan; 7 Liver Cancer Prevention Research Unit, Cluster for Pioneering Research, RIKEN, 2-1 Hirosawa, Wako 351-01, Saitama, Japan

**Keywords:** microfluidic devices, automatic tracking system, eyespot, stigma, flagellum, SM-ZK, *Euglena gracilis*

## Abstract

Cell division of unicellular microalgae is a fascinating process of proliferation, at which whole organelles are regenerated and distributed to two new lives. We performed dynamic live cell imaging of *Euglena gracilis* using optical microscopy to elucidate the mechanisms involved in the regulation of the eyespot and flagellum during cell division and distribution of the organelles into the two daughter cells. Single cells of the wild type (WT) and colorless SM-ZK cells were confined in a microfluidic device, and the appearance of the eyespot (stigma) and emergent flagellum was tracked in sequential video-recorded images obtained by automatic cell tracking and focusing. We examined 12 SM-ZK and 10 WT cells and deduced that the eyespot diminished in size and disappeared at an early stage of cell division and remained undetected for 26–97 min (62 min on average, 22 min in deviation). Subsequently, two small eyespots appeared and were distributed into the two daughter cells. Additionally, the emergent flagellum gradually shortened to zero-length, and two flagella emerged from the anterior ends of the daughter cells. Our observation revealed that the eyespot and flagellum of *E. gracilis* are degraded once in the cell division, and the carotenoids in the eyespot are also decomposed. Subsequently, the two eyespots/flagella are regenerated for distribution into daughter cells. As a logical conclusion, the two daughter cells generated from a single cell division possess the equivalent organelles and each *E. gracilis* cell has eternal or non-finite life span. The two newly regenerated eyespot and flagellum grow at different rates and mature at different timings in the two daughter cells, resulting in diverse cell characteristics in *E. gracilis*.

## 1. Introduction

Cell division is a complex and fascinating process, and prompts researchers to investigate the detailed mechanisms of the process, especially the sequential changes occurring in chromosomes during division. The chromosomes in the nucleus replicate, separate into sister chromatids via the spindle and centrosomes, which results in two new nuclei. The sequential process of nucleus replication has been intensively investigated using optical/electron microscopy and has been elucidated in several eukaryote species. Other self-replicating multiple organelles such as lysosomes, ribosomes, plastids, and mitochondria have also been reported to be equally distributed into two daughter cells during cell division.

Numerous unicellular microbes with nuclei, including yeasts and microalgae, multiply solely via cell division. Some of these microbes possess single organelles, other than the nucleus. For example, the motile microalgae *Euglena gracilis* has an emergent flagellum [1,2] and eyespot (or stigma) [1,3] in each cell, which constitutes single organelle. A simple but essential question arises as to how a single organelle is distributed into two daughter cells during cell division. Two simple possibilities can be conceived: one is that the single organelle is divided into two, and the other is that the original organelle is retained in one daughter cell and a new one is produced for the other daughter cell. It is essential to determine whether one of the two daughter cells receives the organelle from the parental cell as it would affect the life span of the cell. The life span of the cells may be considered “eternal” if both the daughter cells receive newly generated organelles and may be considered “finite” if one of the daughter cells contained the parental organelle and exhibits a shorter life span. The limitation of replicative life span has been reported for individual yeast cells [4], where the mother cell produces daughter cells through bud formation. In this case, only one daughter cell is generated at a time, and the process is relatively easy to track [5]. In contrast, tracking the cell division of *E. gracilis* is challenging. Live imaging at a high magnification is required to track organelles within the cell during cell division because the cells swim as fast as 200 μm/s or more, frequently change their shape, and exhibit euglenoid movements [1,6]. Confinement and fixation of a live cell in between two cover glasses is cumbersome as the cells escape from the observation area with euglenoid movements or are damaged severely by sandwiching pressure from the two glasses.

Non live cell imaging at a high magnification is relatively easier than dynamic live cell imaging and has been extensively carried out using electron microscopy. Walne and Arnott reported the comparative ultrastructure and possible function of eyespots in *E. granulata* and *Chlamydomonas eugametos* [7]. The authors stated that the eyespot granules develop by fusion of smaller granules. Their observations, however, provided only preliminary hints as to how the eyespot develops, to say nothing of the problem of how it replicates during cell division. Osafune and Schiff analyzed the stigma and flagellar swelling in relation to light and carotenoids using transmission electron microscopy [8]. The embedded *E. gracilis* cells were embedded in resin, sectioned, and stained before visualization. They observed that light is required to organize colored carotenoids into the spheres of stigma. Morel-Laurens et al. reported the effects of cell division on the stigma of *Chlamydomonas reinhardtii* and concluded that every daughter cell inherits a portion of the stigma of the maternal cell [9]. However, the mechanisms of stigma division/generation during cell division have not been clarified. Moreover, the mode of cell division differs considerably between *C. reinhardtii* [10] and *E. gracilis* [1].

In the present study, we performed dynamic live cell imaging of *E. gracilis* cell division using optical microscopy. Long-term tracking of a single moving cell was achieved by employing automatic XY-stage control, auto focusing of the microscope, and microfluidic devices for cell confinement. Cell division was recorded on video, and the appearance of the eyespot and the emergent flagellum (extended part out of the anterior of the cell body) was tracked in sequential images extracted from the video. We observed that the eyespot shrank and disappeared at the early stages of cell division. The emergent flagellum was retracted and disappeared before the start of nucleus segmentation. The eyespot remained undetected for approximately 30 min or more, and subsequently two small eyespots appeared and were distributed into two daughter cells. The flagellum was gradually protruded from the anterior portion of each daughter cell after the start of cell cleavage. Our observation revealed that the eyespot of *E. gracilis* is degraded/discarded once during cell division, or at least the carotenoids in the eyespot are extracted/decomposed. The two eyespots are newly reproduced for distribution to the daughter cells.

We also investigated the motility of parental and daughter cells and observed a relatively large difference (an average 18 min in 26 observed cells) in swimming initiation times among the daughter cells after cell division. The results imply that the two daughter cells develop their organelles at different rates, although they each possess each newly formed equivalent eyespot and flagellum.

## 2. Materials and Methods

Two strains of *E. gracilis*, WT Z strain and the colorless mutant SM-ZK [11], were examined in this study. Both strains were cultured at room temperature (23–26 °C) in CM medium [12] with 0.1% ethanol as a carbon source. The cells grew exponentially under heterotrophic conditions under moderate light and atmospheric air without bubbling or shaking. No staining of organelles was performed to avoid cell damage.

**Figure 1:**
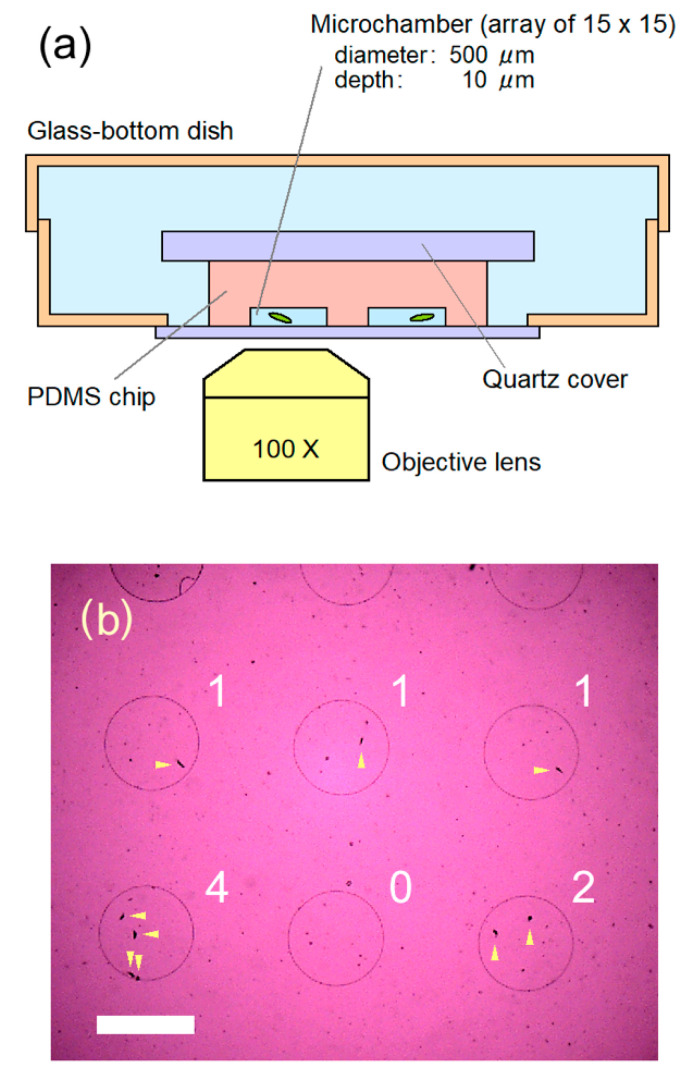
(**a**) Schematic drawing (cross-section, not in scale) of the microfluidic device used in this study. Some of microchambers held a single cell inside, by pressing down the soft porous PDMS chip onto the cell culture solution placed on the glass of the dish. The glass-bottom dish was then filled with pure water to prevent the microchambers from drying. The cells in the closed microchambers can survive three days or more. (**b**) A representative image of cells in confinement in our microfluidic device, observed with an objective lens of 4X. Numbers in the figure show the number of cells confined in each microchamber, and arrows indicate the cells. Scale bar, 500 μm.

Figure 1a shows a schematic illustration of cell confinement in our microfluidic device. The device had a 2-dimensional 15 × 15 array circular microchamber with a diameter of 500 μm and a depth of 10 μm, which was made of porous polydimethylsiloxane (PDMS). A supporting quartz plate (25 mm in diameter, 500-μm thick) was attached on the backside of the PDMS chip (ca. 17 mm square). Fresh CM medium containing 0.1% ethanol (350 μL) was placed on a glass-bottom dish (D910400, Matsunami glass, Osaka, Japan), to which the 200 μL of cell culture was added. The PDMS chip was pressed down onto the solution to confine cells in between the microchambers and bottom glass of the dish. A single cell can be confined by this process in some of the 15 × 15 microchambers, as shown in Figure 1b. After cell confinement, the space inside the glass-bottom dish was filled with pure water to prevent the microchamber from drying. We maintained the microfluidic device containing cells in a resting state for two to three hours before observation to allow the cells to recover from the shock responses caused by confinement. The cells were able to survive for more than three days in the microchambers.

**Figure 2:**
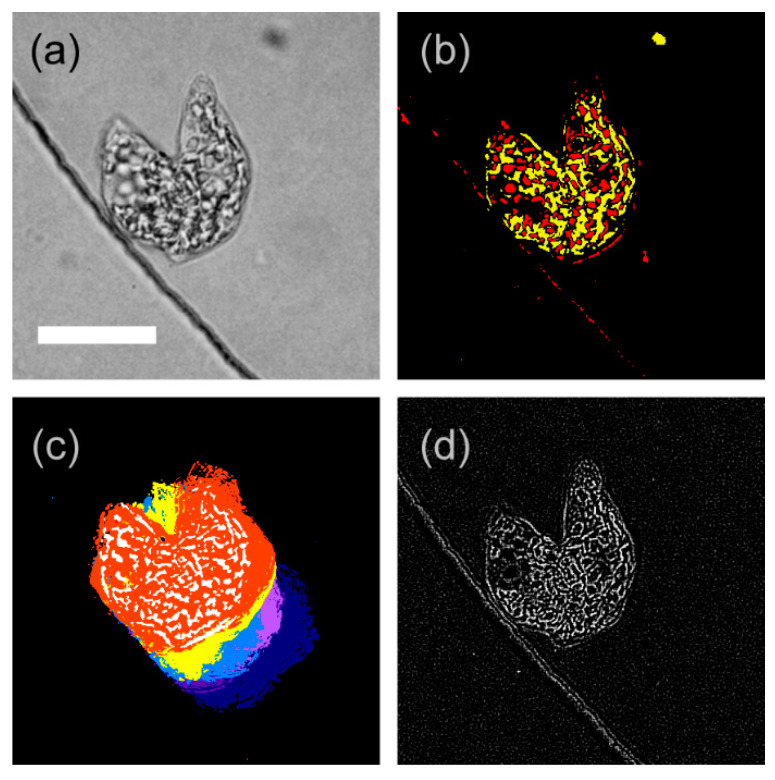
Examples of image processing for cell tracking, wherein a cell in the later stage of cell division at the edge line of circular microchamber was processed. (**a**) Captured image; obtained by 3 × 3 mean filtering with gray scaling. (**b**) Contrast image; obtained by thresholding brightness deviated from whole area average, i.e., the brighter part is colored in red, whereas the darker part is colored in yellow. (**c**) Trace image; obtained by time differential (frame-by-frame differential for each pixel) of the captured image, with further processing of binarization and integration for 20 frames (5.3 s). Five trace images were superimposed with different colors for effective visualization. (**d**) Focusing image; obtained by the spatial derivative for 3 × 3 pixels on the captured image. Scale bar, 20 μm.

Our cell imaging system was based on an inverted optical microscope (IX71, Olympus, Tokyo, Japan) equipped with an auto XY-stage (BIOS-206T-OL, Sigma Koki, Tokyo, Japan) and controller (FC-101G, Sigma Koki, Tokyo, Japan). For automatic focusing, we attached a stepping motor and its controller (RZC001, Kyowa Denshi, Tsukuba, Japan) to the focusing knob of the microscope. The selected microchamber was observed under an 100X oil-immersion objective lens (UPlanFLN 100X/1.30, Olympus, Tokyo, Japan) with transmitted illumination of a halogen lamp through a condenser lens. The illumination intensity measured at the observation area was 4.3 mW/cm^2^ (500 nm equivalent, corresponding to approx. 170 μmol/m^2^s), which did not induce a photophobic response on *E. gracilis* [13,14,15]. The observed image was split into two paths; one path for monitoring with a CMOS camera (IUC-200CK2, Trinity, Gunma, Japan) and the other path for video recording (HDR-XR550, Sony, Tokyo, Japan). The images captured with the CMOS camera were transmitted to a PC (MG/D70, Fujitsu, Tokyo, Japan), and processed with an original algorithm for cell tracking. The captured image (Figure 2a) of 6.8 pixel/μm resolution was converted into a contrast image based on brightness deviation from the whole area average (Figure 2b), a trace image based on frame-by-frame differentiation/binarization/integration (Figure 2c), and a focusing image based on second derivative (Figure 2d). When the cell movement was clearly observed in the trace image, the algorithm sent a signal to the XY-stage to follow the center of the trace. If movement was not detected clearly, the algorithm followed the center of the extracted image of the cell. When the cell was missing in both the trace cell-extracted images, the algorithm starts to search the cell with a random walk. The algorithm also periodically fine-tunes the focus of the lens by stepwise movement and selecting the most adequate focusing point. Our system enables us to track the cell in the microchamber automatically for more than three days with continuous video recording of the observation area. Detailed images were obtained from the video recording after the termination of the experiment.

We also elucidated the motility of parental and daughter cells under a long-term millimeter-scale observation. Square microchambers with a side length of 2.5 mm and a depth of 100 μm [16] were used with a 5X objective lens (MPlanFLN 5X/0.15, Olympus, Tokyo, Japan). We confined a single SM-ZK cell in the microchamber and tracked its swimming traces to measure the time point at which the parental cell stopped swimming to initiate cell division (T1), and the time point at which the two daughter cells started swimming after cell division (T2, T3). The non-swimming period (T2 − T1) represents the duration of cell division, whereas difference in swimming start times (T3 − T2) represents the growth difference between two daughter cells.

All the above experiments were performed at room temperature (23–26 °C) in CM medium with 0.1% ethanol as a carbon source.

## 3. Results

### 3.1. Outline of Cell Division in Microchamber

**Figure 3:**
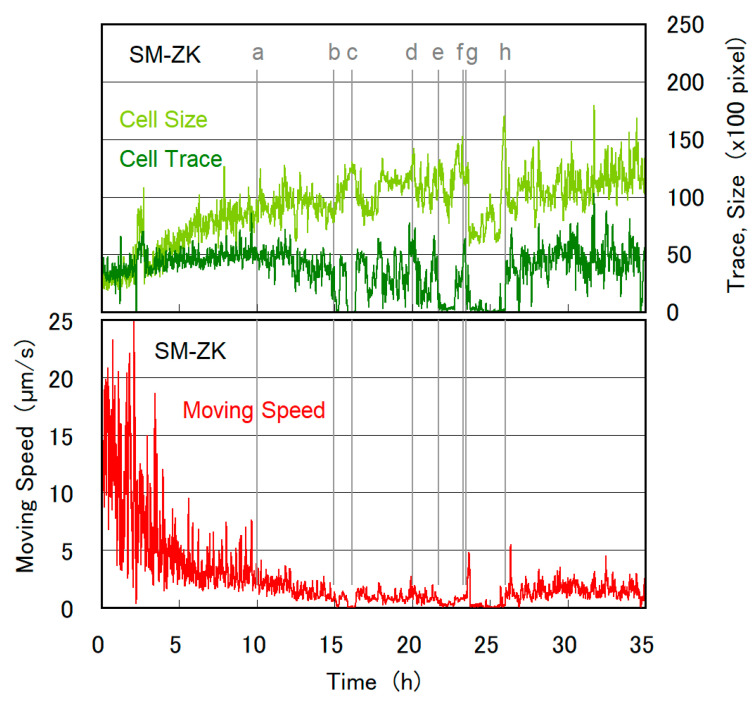
Temporal evolution of moving speed (velocity), cell size, and trace value obtained during the time course of cell division for an SM-ZK cell. Moving speed was calculated from XY-stage movement and the center of the gravity of the contrast image (e.g., Figure 2b). Cell size was obtained by counting red/yellow pixels in each contrast image (e.g., Figure 2b). The cell size represents the quasi-2D (10 μm thick) volume of the cell, assuming that cell height is at a uniform height of 10 μm. Trace value was obtained by estimating the non-zero pixels (white and red) in the trace image (e.g., Figure 2c). The trace value represents the momentum of cell movement and activity inside the cell. When the cell stops moving, the moving speed drops to zero, whereas the trace value may not become zero if the organelles move inside the cell. All of three values in Figure 3 were time-averaged for 2 min. The contrast and trace images for (a) to (h), and video-recorded images are given in Figure 4 and Figure 5, respectively.

**Figure 4:**
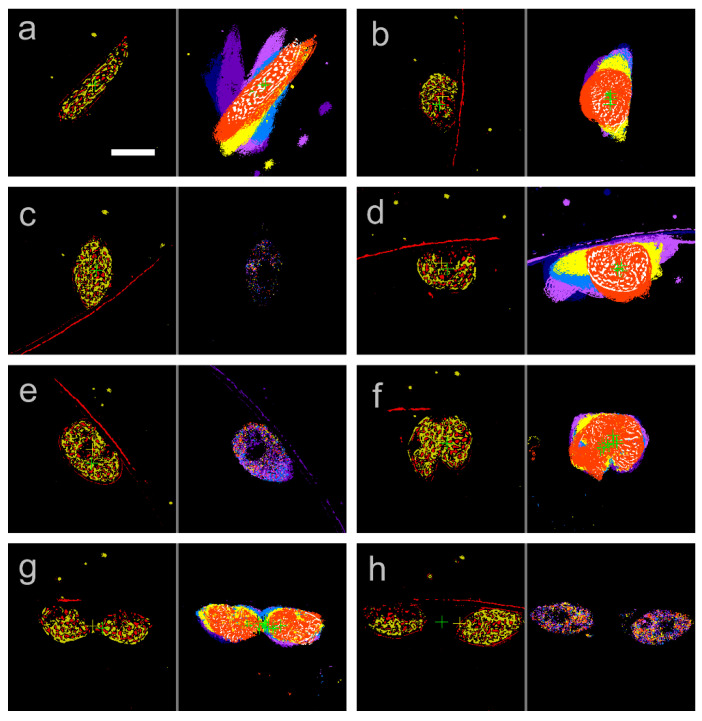
Contrast (**left**) and trace image (**right**) observed at (**a**) 10.0 h, (**b**) 15.0 h, (**c**) 16.1 h, (**d**) 20.0 h, (**e**) 21.7 h, (**f**) 23.3 h, (**g**) 23.5 h, and (**h**) 26.0 h. The time point of each image is given in Figure 3 as vertical lines. Cross marks in the images are the center of gravity of non-zero pixels for the purpose of XY-stage control. Scale bar, 20 μm.

Dynamic live cell imaging is more advantageous than non-live cell imaging with electron microscopy as it tracks the whole cell division process in a single cell. Figure 3 shows long-term in situ tracking of the moving speed, cell size, and trace value of a single SM-ZK cell confined in the microchamber during the cell division period. Cell size was measured as non-zero pixels in a contrast image (Figure 2b), which represents the quasi-2D (10 μm thick) volume of the cell. Trace value was measured as non-zero pixels in the trace image (Figure 2c), which represents the total cellular movement, i.e., cell locomotion, shape change, and the movement of cellular components inside the cell. As shown in Figure 3, for 0–10 h, the speed of the cell gradually decreased to ca. 2.5 μm/s, whereas the cell size increased to approximately three times. The velocity of the cell was considerably lower than that observed for SM-ZK cells in free water, which reached up to ca. 200 μm/s or more. As the thickness of the microchamber was only 10 μm, the movement of the flagellum was limited, and the friction between the cell body and microchamber ceiling/floor increased with cell size. The cell shape was a spindle at 10 h, as shown in Figure 4a. Increase in cell size and decrease in velocity continued till 15 h (Figure 3), whereas the cell shape changed between a fat spindle and a sphere at a higher frequency (Figure 4b). At around 16 h, velocity and trace value suddenly fell to almost zero. Fewer traces were observed in the trace image, whereas the contrast image remained clear (Figure 4c). Thus, the results indicate that the cell stopped moving and organelle movement inside the cell also stopped, which are typical signs of the initiation of cell division. However, the cell did not enter into cell division at this time and resumed its motion after ca. 30 min of being motionless. For 16.4–21.7 h, the cell behavior was almost the same as that observed before 15 h (Figure 4d), with occasional short motionless breaks. At ca. 21.7 h, the cell became motionless again as shown in Figure 4e. After this stage, cell division proceeded, with the cell initiating cleavage at 23.3 h (Figure 4f). As the cell altered its shape continuously during cleavage, the trace value became higher as seen in Figure 3 at 22.8–23.7 h. Cell cleavage was almost completed at 23.5 h, as two daughter cells were observed (Figure 4g). One of the daughter cells migrated outside the observation area, resulting in a drop in cell size at 23.7 h. The two daughter cells returned to the observation area again at 25.7–26.2 h, causing a sudden spike in cell size. The daughter cells grew in size after 23.5 h (Figure 3), with occasional periods of no motion (Figure 4h).

The cell division observed in Figure 3 was one of the typical cases, and the initiation of cell division differed cell to cell. However, motionless periods appeared at the beginning of cell division for all cells studied. The outline of cell division was same for WT cells (Supplementary Figure S1). It is noteworthy that the cell division of *E. gracilis* is closely related with its movement, i.e., the mitotic cell ceased euglenoid movements as well as swimming. This observation implies that the reproduction and reconfiguration of organelles persists during the motionless period; i.e., cells are unable to swim or migrate with euglenoid movements at the initial stage of cell division. Additionally, we found that 20 μm in the depth of the microchamber was too large to cover the whole cell within the focusing depth of the 100× objective lens, whereas 5 μm was too small for cell division. A cell in a 5 μm deep microchamber was pressed to a flat conformation, remained motionless, and failed to start cell division. The suppression of cell division in a 5 μm deep microchamber revealed that the cell must form a spherical shape to dynamically reconfigure organelles inside the cell. The functional importance of mitotic rounding has been reported as that it influences spindle assembly and is required for a successful cell division [17].

### 3.2. Disappearance of the Eyespot and Flagellum during Cell Division

The appearance of the eyespot under an optical microscope is distinctive, as proved by the fact that the name *Euglena* is a combination of the Latin words eu (beautiful) and glena (eye), invented by Dutch scientist Antonie van Leeuwenhoek [18]. As shown in supplementary Figure S2, the eyespot located in the anterior part of a non-mitotic cell can be observed as a bright/dark red sphere (Figure S2a,b), red rod/crescent (Figure S2c), or dark gray sheet (Figure S2d), as it varies with the posture/shape of the cell. The eyespot in *E. gracilis* is composed of several spherical globules (Figure S2a), in which carotenoids are stored [19]. Kato et al. revealed that the eyespot plays an important role in the photoresponses of *E. gracilis*, i.e., the initiation of phototaxis and the shadowing of photoreceptor [20].

**Figure 5:**
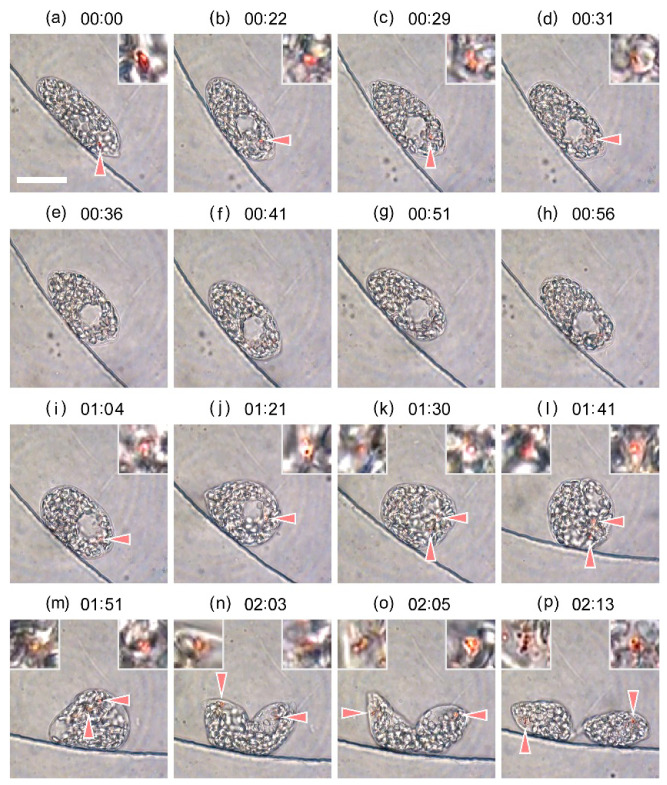
Images extracted from the video recording of the experiment depicted in Figure 3. (**a**) 21.24 h, (**b**) 21.60 h, (**c**) 21.72 h, (**d**) 21.75 h, (**e**) 21.83 h, (**f**) 21.92 h, (**g**) 22.08 h, (**h**) 22.17 h, (**i**) 22.30 h, (**j**) 22.58 h, (**k**) 22.73 h, (**l**) 22.92 h, (**m**) 23.08 h, (**n**) 23.28 h, (**o**) 23.32 h, and (**p**) 23.46 h. Time index given in the figure is shifted as reference; 00:00 (hh:mm) corresponds to 21.24 h in Figure 3. The eyespot indicated by arrow is enlarged by four times in the inset. Scale bar, 20 μm. Dynamic evolution of cell division is provided in the supplementary movie S3.

Figure 5 shows detailed images obtained from the representative experiment shown in Figure 3, for the cell division duration of 21.24–23.46 h. The time index for each image was shifted for simplicity; 00:00 (hh:mm) corresponds to 21.24 h in Figure 3. A majority of granules observed in the images were those storing paramylon starch. The nucleus and reservoir located in the anterior part of the cell were observed as large transparent ellipsoidal spheres. Before the cell ceased moving at 21.7 h (Figure 4e), the eyespot was clearly observed near the anterior of the cell (Figure 5a). After 21.7 h, the nucleus stayed at the anterior center of the cell, observed as a large hole in the images. The eyespot gradually diminished in size (Figure 5b–d) and disappeared at 21.83 h (Figure 5e). This process continued for 45 min or less. The eyespot remained undetected for ca. 30 min (Figure 5e–h), and subsequently a small young eyespot appeared (Figure 5i) at 22.30 h. The eyespot grew slowly, while the second eyespot could not be seen for the next 25 min (Figure 5j). It appeared as if two new eyespots started growing individually at distinct rates. At 22.73 h, the segmentation of nucleus began, and two small balls of eyespot were simultaneously observed (Figure 5k). Nucleus segmentation was completed at ca. 22.78 h. After 22.92 h, two young identical eyespots were observed (Figure 5l–p). Cell cleavage started at 23.17 h, and was almost completed at 23.46 h (Figure 5p). The two eyespots grew slowly and matured by 23.46 h, as suggested by small ball-like composites observed in Figure 5o,p.

**Figure 6:**
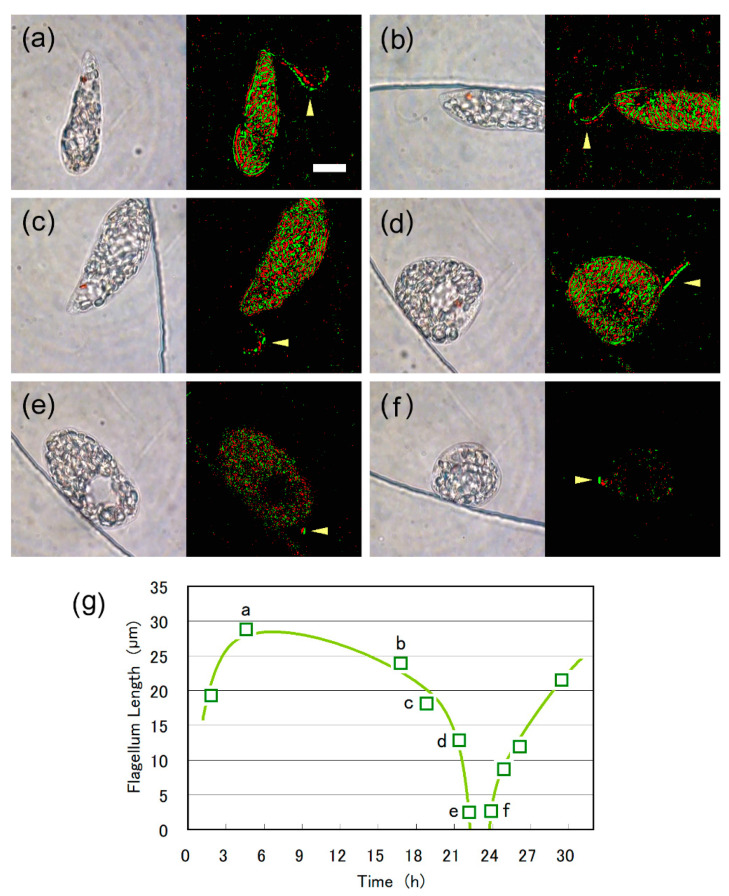
Shortening of the emergent flagellum, as seen in the images extracted from the video recording (**left** panels) and enhanced images (**right** panels) of the Figure 5 case. Enhanced images were obtained with differentiation and binarization of two images at 61 ms intervals. The length of the emergent flagellum was (**a**) 29 μm at 4.57 h, (**b**) 24 μm at 16.78 h, (**c**) 18 μm at 18.83 h, (**d**) 13 μm at 21.41 h, (**e**) 2.5 μm at 22.18 h. (**f**) After the cell division, the emergent flagellum (2.6 μm in length) was first observed for one of the daughter cells at 23.91 h. Scale bar, 10 μm. (**g**) Temporal change in flagellum length. The graphical line is provided to indicate the trend.

The above observation can be visualized in the supplementary movie S3. The cell frequently altered between spindle and ball shape before 00:35 h in the time index. A shrinking eyespot was observed among the paramylon granules at this period, as well as moving flagellum at the anterior end of the cell. For the time period between 00:35 h and 00:57 h, the cell mostly halted its shape alterations, and no trace of eyespot was observed. The emergent flagellum was shortened and finally disappeared at 01:07 h. The shortening of the emergent flagellum was clearly visualized by enhancing the extracted images with short-period (61 ms) differentiation and binarization as shown in Figure 6. The length of the emergent flagellum was 29 μm at 4.57 h, whereas it reduced to 2.5 μm at 22.18 h (00:56 h in Figure 5 and Movie S3). The shortening of emergent flagellum was accelerated after 18h as shown in Figure 6g. No emergent flagellum was detected after 22.20 h (00:57 h) even with the enhancement of extracted images. After the cell division, a new emergent flagellum, 2.6 μm in length, was first observed for one of the daughter cells at 23.91 h, which subsequently grew over 20 μm in length (Figure 6g).

Note that the disappearance of the eyespot and the emergent flagellum elucidated in the present study does not imply that these organelles were completely degenerated to molecular levels. We show that the eyespot of *E. gracilis* is decomposed before the nucleus segmentation such that it is not visible under an optical microscope, and that the emergent part of the flagellum is retracted into the cell body. Furthermore, we show that these organelles are regenerated during cell division for the two daughter cells.

A dark red spot was observed after 00:57 h, which gradually formed an eyespot. The development of the other eyespot was not clear, and the two eyespots were not observed distinctively until 01:30 h. The cell became round at 01:23 h and the nucleus began segmentation at ca. 01:29 h. After the cell cleavage starting at 01:57 h, two eyespots were observed at the two anterior regions of cleaved cells. A flagellum was not observed for each of the daughter cells at the end of movie S3 (02:13 h), which initially appeared as a short string at 02:20 h (23.57 h) as shown in Figure 6f. A few straying black spots or small white spheres were observed around the nucleus occasionally in Movie S3 for the entire period between 00:00 h and 02:13 h, which might be the one reported as reserve or waste globules [21].

**Figure 7:**
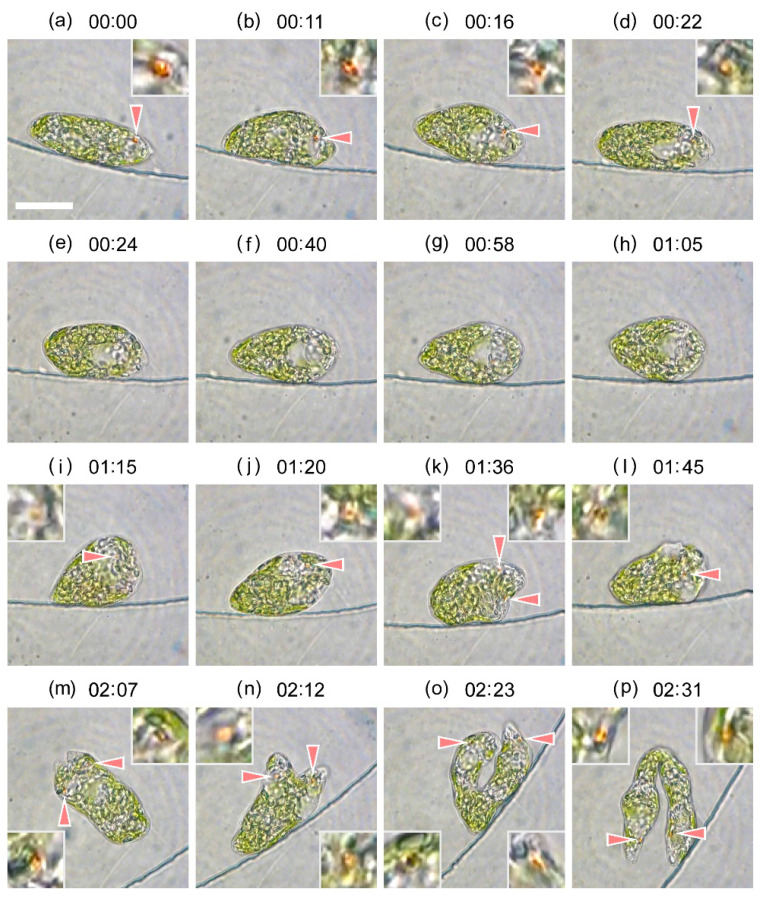
Images extracted from the video recording of the experiment depicted in Figure S1. (**a**) 17.52 h, (**b**) 17.70 h, (**c**) 17.79 h, (**d**) 17.89 h, (**e**) 17.92 h, (**f**) 18.19 h, (**g**) 18.49 h, (**h**) 18.60 h, (**i**) 18.77 h, (**j**) 18.85 h, (**k**) 19.12 h, (**l**) 19.27 h, (**m**) 19.64 h, (**n**) 19.72 h, (**o**) 19.90 h, and (**p**) 20.04 h. Time index given in the figure is shifted as a reference; 00:00 (hh:mm) corresponds to 17.52 h in Figure S1. The eyespot indicated by arrow is enlarged by four times in the inset. Scale bar, 20 μm. Dynamic evolution of cell division is provided in the supplementary movie S4.

Similar observations were obtained for WT cells as shown in Figure 7 and supplementary movie S4. The eyespot was slightly hard to see due to the presence of green plastids. In the case of Figure 7 (Movie S4), the eyespot was not observed for ca. 50 min (00:24–01:15 h in movie S4), and the emergent flagellum disappeared for 45 min (00:45–01:30 h). The reservoir was clearly observed until 01:00 h, however, only the nucleus was observed after 01:10 h. Nucleus segmentation took place at 01:15 h, and a new eyespot appeared. Cell cleavage started at 01:30 h, and a new flagellum emerged. The daughter cells were considerably elongated compared to that of SM-ZK cells (Figure 5, Movie S3), and cell separation prolonged.

**Figure 8:**
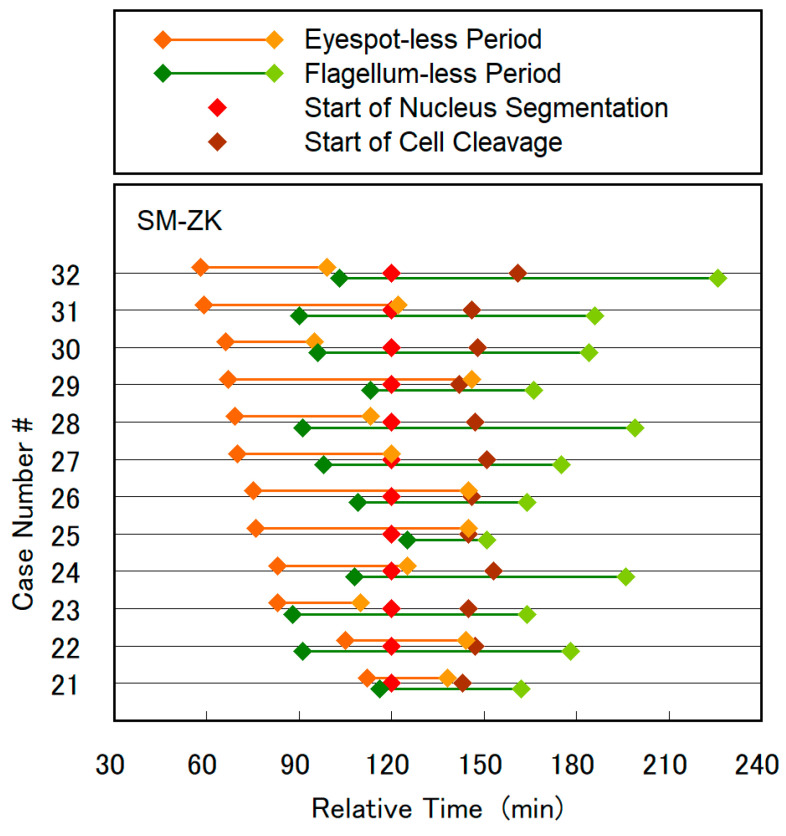
The period of disappearance of the eyespot and flagellum plotted for 12 SM-ZK cells, together with the initiation time of nucleus segmentation and cell cleavage. In the plot, the initiation time of nucleus segmentation was used as the standard timing (120 min). Case number was sorted by the time of eyespot disappearance (earlier to later). The case #30 corresponds to Figure 3, Figure 5 and Figure 6, and Movie S3. See Figure S5 for WT cells.

The period of eyespot and flagellum disappearance was plotted for 12 and 10 distinct cells of SM-ZK in Figure 8 and WT in supplementary Figure S5, respectively, together with the initiation time of nucleus segmentation and cell cleavage. In these plots, the initiation time of nucleus segmentation was used as the standard timing (120 min) for reference. The plots showed a scattered distribution due to diversity in cell characteristics as well as difficulty in the observation of small organelles. However, important trends and statistics could be deduced from the plots. In most of the cells of the SM-ZK and WT strains, the disappearance/reappearance of the eyespot was antecedent to the disappearance/reappearance of the flagellum. The eyespot disappeared for 8–62 min (43 min on average, 17 min in deviation) prior to nucleus segmentation in SM-ZK, and the eyespot-deficient period remained for 26–79 min (48 min on average, 18 min in deviation). The discernible eyespot reappears prior to the initiation of cell cleavage, and in some cells, even prior to the initiation of nucleus segmentation. Similar results were obtained for WT as shown in the Figure S5 caption. The flagellum disappeared at 0–0.5 h before nucleus segmentation and reappeared after ca. 1.0–2.0 h of its absence. This occurred mostly 0.5–1.0 h after the initiation of cell cleavage. Figure 8 and Figure S5 clearly show that the eyespot is decomposed and the emergent flagellum is shortened at cell division, and two daughter organelles are newly formed, for both SM-ZK and WT strains.

### 3.3. Cell Motility Observed on a Large Scale

**Figure 9:**
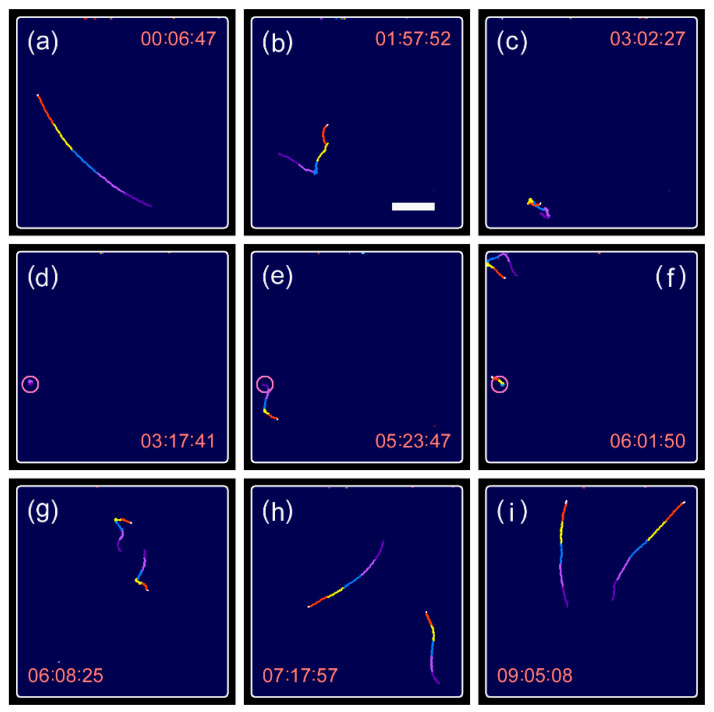
Swimming traces observed in a long-term millimeter-scale observation, including the period of cell division. (**a**–**d**) Movements of the parental cell, (**e**–**i**) movements of the daughter cells. In the images shown in (**a**–**i**), the traces obtained for the last 12.3 s (5 frames × 2.47 s/frame) were superimposed with different colors for visualization. Circles in (**d**–**f**) show the location where the cell division occurred. Refer to supplementary movie S6 for visualizing the entire process. Time index in the panels is indicated by hh:mm:ss. Scale bar, 500 μm.

It is important to assess whether the two daughter cells grow at an equal rate after cell division. This was determined by a long-term millimeter-scale observation of swimming motion before and after cell division. A typical example of swimming traces obtained in the long-term millimeter-scale observation is presented in Figure 9 and Supplementary Movie S6. The cell was swimming in a straight direction at a speed of ca. 160 μm/s during interphase (Figure 9a). Approximately two hours prior to cell division, irregular swimming traces were observed with occasional random turns (Figure 9b), with an increasing frequency of turns. The cell showed the irregular swimming pattern continuously with slowed down speeds as cell division approached (ca. 30 min before) (Figure 9c). Cell division started (Figure 9d) after several times of short-lived instances of no movement (supplementary movie S6), which corresponds well with the observation of Figure 3 and Figure 4. The first daughter cell started swimming 126 min after cell division started, whereas the second daughter cell remained motionless at the place of the cell division (Figure 9e). The second daughter cell started swimming after a delay of 38 min (Figure 9f). The two daughter cells showed irregular swimming traces ca. one hour after the initiation of swimming (Figure 9g), and gradually recovered to swimming in a straight direction (Figure 9h) at an increasing speed (Figure 9i).

**Figure 10:**
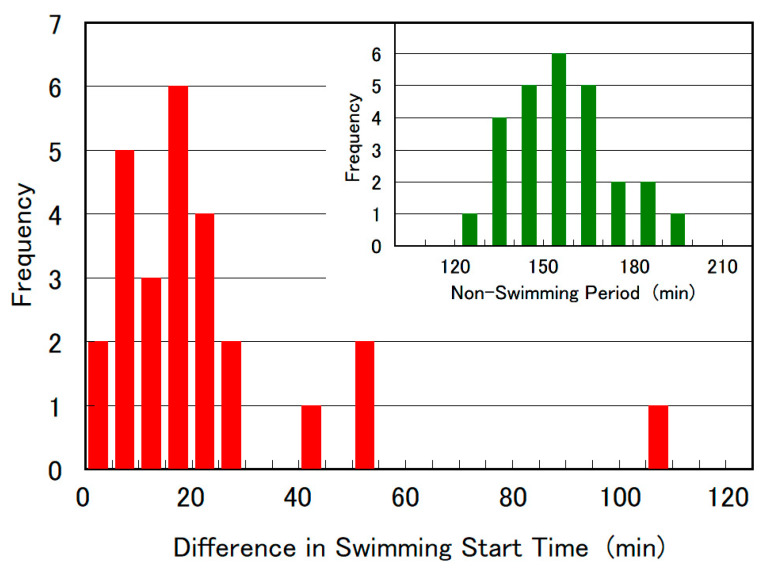
Histogram of the duration of cell division (non-swimming period) and difference in swimming start time between two daughter cells, based on data obtained from 26 individual SM-ZK cell divisions. The average cell division duration and difference in swimming start time was 157 and 38 min, respectively.

Figure 10 shows the histogram of cell division time (the duration the cell remained motionless during long-term observation) and the difference in swimming start time between two daughter cells, based on data from 26 individual instances of SM-ZK cell division. The average duration of cell division and difference in swimming start times were 157 and 38 min, respectively, and no correlation was found between these two parameters.

## 4. Discussion

Although there are several studies on the cell division of *E. gracilis*, a majority of them focus on nucleus segmentation with a few describing eyespot behavior. Buetow reviewed the morphology and ultrastructure of *Euglena* [1] and reported that the stigma of *E. gracilis* is composed of many spherical granules (diameter of 100–300 nm), which are present in the cytoplasm in a loosely packed group [22,23]. The review also pointed out that the eyespot is a functionally specialized organelle, and that it is self-reproducing and occupies a defined portion of the cytoplasm. Leedale analyzed the mitotic cycle in live *E. gracilis* cells under an optical microscope, and showed that the locomotor apparatus (flagella, photoreceptor, and eyespot) replicates and the reservoir divides during early-to-late metaphase succession [24]. Hall and Jahn [25,26], and also Gojdics separately [27], reported that eyespot breaks up into its component granules during cell division at the stage of flagellum duplication or nucleus migration, with optical microscopy, although no evidential image showing eyespot break up was presented in their reports. Gillott and Triemer reported on the ultrastructure of *E. gracilis* at different stages of mitosis from preprophase to telophase, as observed under an electron microscope [28]. The only remark on the flagellum or eyespot found in the literature was that four non-emergent flagellar cross-sections were observed in a cell at prophase. Based on electron micrographs, Kivic and Vesk revealed that the eyespot replicated after nuclear division and segregated to opposite sides of the reservoir at about the same time as the flagella duplicated [19,21]. These observations and findings were, however, rather fragmented and non-qualitative, and observed in fewer cases. The dynamic live cell imaging of *E. gracilis* cell division in the present study will offer insight into the replication processes of the eyespot and flagellum, as well as the mechanisms required for their reproduction.

Dynamic live cell imaging of cell division revealed that the eyespot of *E. gracilis* is removed/discarded once during cell division by a gradual shrinking process, or possibly the carotenoids in the eyespot are extracted/decomposed. We have not observed the break up of eyespot into a number of small globules in our dynamic live cell imaging of cell division, as suggested by the former literature [25,26,27]. On the other hand, Kato et al. reported that their genetically modified (Eg*crtB*-suppressed) colorless strain of *E. gracilis* had no obvious red eyespot but still possessed empty eyespot globules [20]. The report implies that the eyespot reproduction proceeds in two steps; the membranes of eyespot globules are reproduced, and then carotenoids accumulate into the globules. Figure S7a shows an example before the reappearance of the new distinctively discernible eyespot (after nucleus segmentation and before cell cleavage), in which a reddish brown hazy region can be seen between the two nuclei. This may present newly synthesized carotenoids, which will eventually be loaded into the eyespot globules.

The shortening of the emergent flagellum of *E. gracilis* is also the key for motility during cell division, i.e., the cell cannot swim due to the lack of emergent flagellum. The remaining issue in the flagellum shortening is whether the flagellum is decomposed from its outer end point or is retracted inside the reservoir from the backend. During the shortening process, a white dim spot was often observed at the outer end point of the flagellum (as shown in Figure S7b) even for extremely short flagellum of almost zero length. Although the origin of the white spot has not been identified, our observation suggests that the flagellum is retracted inside the reservoir, whereas the outer end point remains unchanged. The retraction of emergent flagellum during cell division has been also reported for *C. reinhardtii* [29].

The reason why *E. gracilis* degrades the eyespot and flagellum and regenerates them (instead of generating a new organelle and distributing new and old ones into two daughter cells) still remains enigmatic. We consider that the organellar decomposition/reproduction function of the organism is more basic and advantageous than that of division and distribution. Degradation of organelles is a basic function of cells and is required for the removal of unnecessary and defective cytosolic components. Regeneration of organelles is also frequently observed in microbes, as evidenced by the observation that *E. gracilis* cells regenerate their flagella once it is lost by external stimuli [30,31]. Beech et al. reviewed instances of flagellum duplication inside the basal body of algal flagellates, which included observations of flagellum shortening (*Epipyxis*) or complete retraction (*Chlamydomonas*, *Pleurochrysis*) during mitosis [32].

Our observation revealed that each of the two daughter cells of *E. gracilis* possess a newly replicated eyespot and emergent flagellum, and therefore the life span of *E. gracilis* may be “logically eternal.” At the same time, during the process of cell division shown in Figure 5, the two new eyespots were not generated simultaneously. One eyespot appeared approximately 25 min before the appearance of the second one (Figure 5i,k). Additionally, the two daughter cells seemed to grow at different rates after cell division, as evidenced by a relatively large difference in swimming start time (18 min on average, Figure 10). These observations suggested that the two newly regenerated eyespot and flagellum may grow at different rates and mature at different timings, possibly due to unequal distribution of cytoplasm or stored nutrients in the two daughter cells during cell division. These differences produce diverse cellular characteristics of *E. gracilis*, such as in their motile behavior of gravitaxis [16], chemotaxis [33], and phototaxis [13,14,15]. Further investigation on cell division and maturity is required to explain this phenomenon in *E. gracilis* and other algal flagellates.

WT and SM-ZK cells contain a number of large paramylon granules, which tend to hinder the detection of the eyespot and detailed tracking of the shrinking eyespot. Upon suppression of paramylon production in SM-ZK cells using RNA interference or genetic modification, the eyespot reproduction process can be elucidated in detail using optical microscopy. The carotenoid specie present in the eyespot globules has been identified by Tamaki et al. as zeaxanthin [34]. Upon realization of fluorochrome-labeling of zeaxanthin molecules, fluorescence microscopy may be employed to track fluorochrome-labeled zeaxanthin molecules and analyze the replication process. Advanced laser processing method, such as eyespot destruction by focused femto-second laser pulses on live *E. gracilis* cells [35], is also a promising approach to investigate eyespot self-reproduction. A mutant *Euglena* strain containing plastids but no eyespot may be generated when eyespot regeneration is eliminated by advanced laser processing.

## 5. Conclusions

Our dynamic live cell imaging for 12 SM-ZK cells and 10 WT cells revealed the following. (1) The eyespot of *E. gracilis* was degraded once during cell division, or the carotenoids in the eyespot were decomposed. This process occurs predominantly 0.5–1.0 h prior to nucleus segmentation. (2) Two eyespots are gradually formed after ca. 0.5–1.5 h of its absence, and 0–0.5 h before the initiation of cell cleavage. (3) The emergent flagellum is retracted to zero-length 0–0.5 h before nucleus segmentation. (4) Two flagella gradually emerge 0.5–1.0 h after the initiation of cell cleavage (1.0–2.0 h of absence). (5) The newly regenerated eyespot and flagellum may grow and mature at different rates in the two daughter cells. Based on our microscopic observations, it is reasonable to assume that the two daughter cells are identical in terms of organelle age. These results also do not refute the hypothesis that the life span of *E. gracilis* may be “logically eternal,” although further studies are required to confirm it. The dynamic live cell imaging technique demonstrated in this study is an efficient approach to investigate organelle regeneration in unicellular microbes, especially on genetically modified strains of *E. gracilis* and other microalgae.

## Data Availability

Data is contained within the article or supplementary materials.

## Supplementary Materials

The following are available online at https://www.mdpi.com/article/10.3390/plants10102004/s1. Figure S1: Temporal evolution of moving speed (velocity), cell size, and trace value obtained during the time course of cell division for a WT cell. All three values were time-averaged for 2 min. Cell division was observed for the duration of 17.8–20.3 h. Similar trends to Figure 3 can be observed for three curves. The video-recorded images for (a) to (h) are given in Figure 5. Figure S2: Variation of eyespot appearance for a non-mitotic SM-ZK cell; observed as a small red ball composite (a), dark red sphere (b), red rod (c), and dark gray sheet (d). Scale bar, 20 μm. Movie S3: Cell division observed for an SM-ZK cell in the microchamber. The movie was reproduced in a time-lapse manner from the original video-recording, with a 41 times higher playback speed. Slow frame advance playback is recommended. Time stamp in the movie represents hh:mm. Scale bar, 10 μm. Movie S4: Cell division observed for WT cell in the microchamber. The movie was reproduced in a time-lapse manner from the original video-recording, with a 41 times higher playback speed. Slow frame advance playback is recommended. Time stamp in the movie represents hh:mm. Scale bar, 10 μm. Figure S5: The period of disappearance of the eyespot and flagellum plotted for 10 WT cells, together with the initiation time of nucleus segmentation and cell cleavage. In the plot, the initiation time of nucleus segmentation was used as the standard timing (120 min). The eyespot disappeared 44–70 min (78 min in average, 15 min in deviation) before the nucleus segmentation for WT, and eyespot-less period remained for 51–97 min (48 min in average, 18 min in deviation). The case #4 corresponds to Figs. S1 and 7 and movie S4. Movie S6: Swimming traces observed under a long-term millimeter-scale observation, including the period of cell division. The time index in the movie is provided in the format hh:mm:ss. The parental cell stopped swimming at 03:17:53, and cell division occurred at the location marked by a circle. The first daughter cell started swimming at 05:23:47, and thus the duration of cell division in this movie was 126 min. The second daughter cell started swimming at 06:01:50, and thus the difference in swimming start times between the first and second daughter cell was 38 min. Figure S7: (a) Reddish brown hazy region (encircled) observed before the reappearance of a new distinctive eyespot (after the nucleus segmentation and before cell cleavage). (b) White dim spot (indicated by an arrow) observed at the outer end of shortened flagellum. Scale bar, 10 μm.
