# Supplementary material for: Regeneration of the Eyespot and Flagellum in Euglena gracilis during Cell Division
Source: Plants (Basel). 2021 Sep 24;10(10):2004. doi: 10.3390/plants10102004 (PMC8537169; doi:10.3390/plants10102004)
Supplement: Supplementary file 1 [file plants-10-02004-s001.zip › supplementary files 1390944.pdf]

Supplementary Figures with captions

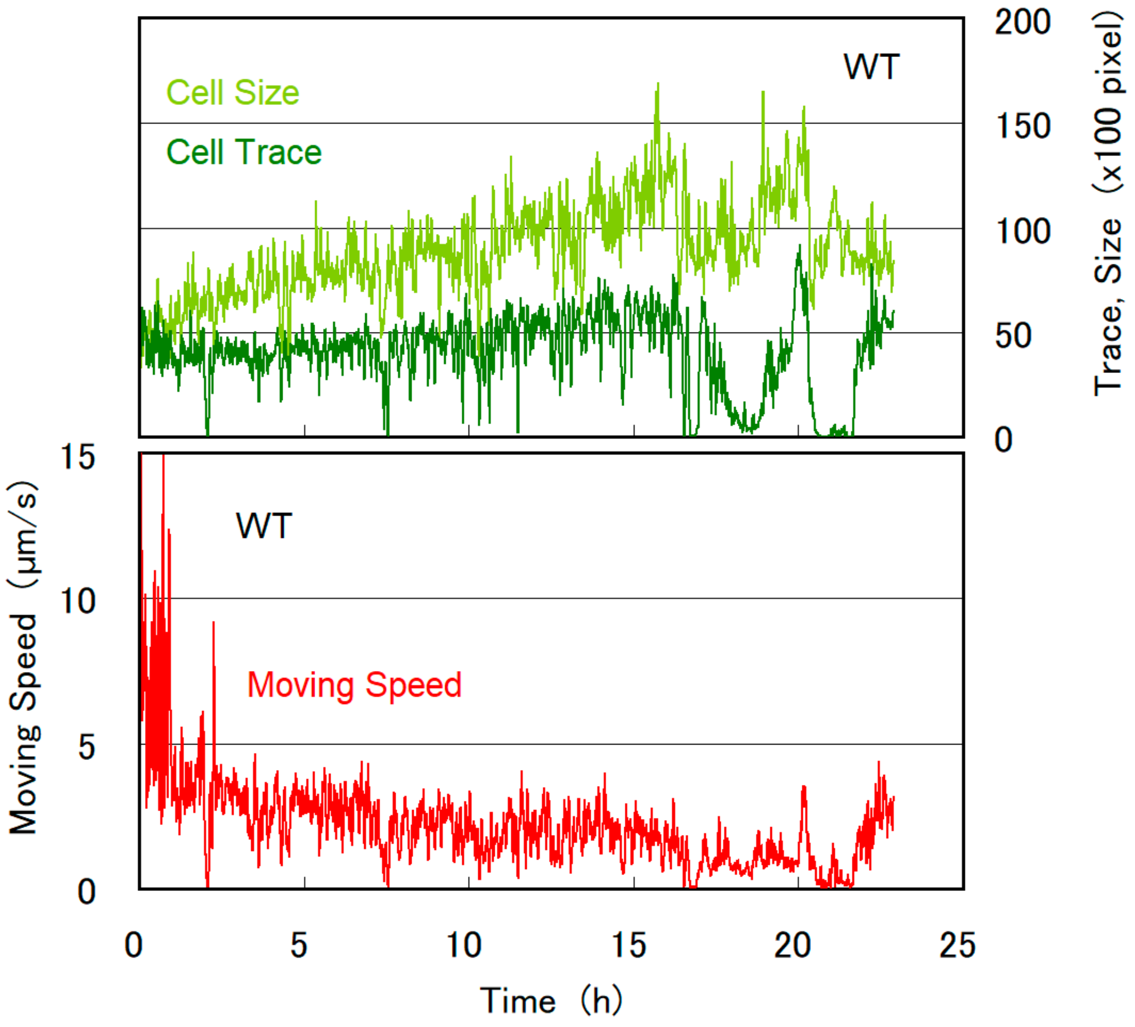

Figure S1. Temporal evolution of moving speed (velocity), cell size, and trace value obtained during the time course of cell division for a WT cell. All three values were time-averaged for 2 min. Cell division was observed for the duration of 17.8–20.3 h. Similar trends to Fig. 3 can be observed for three curves. The video-recorded images for (a) to (h) are given in Fig. 5.

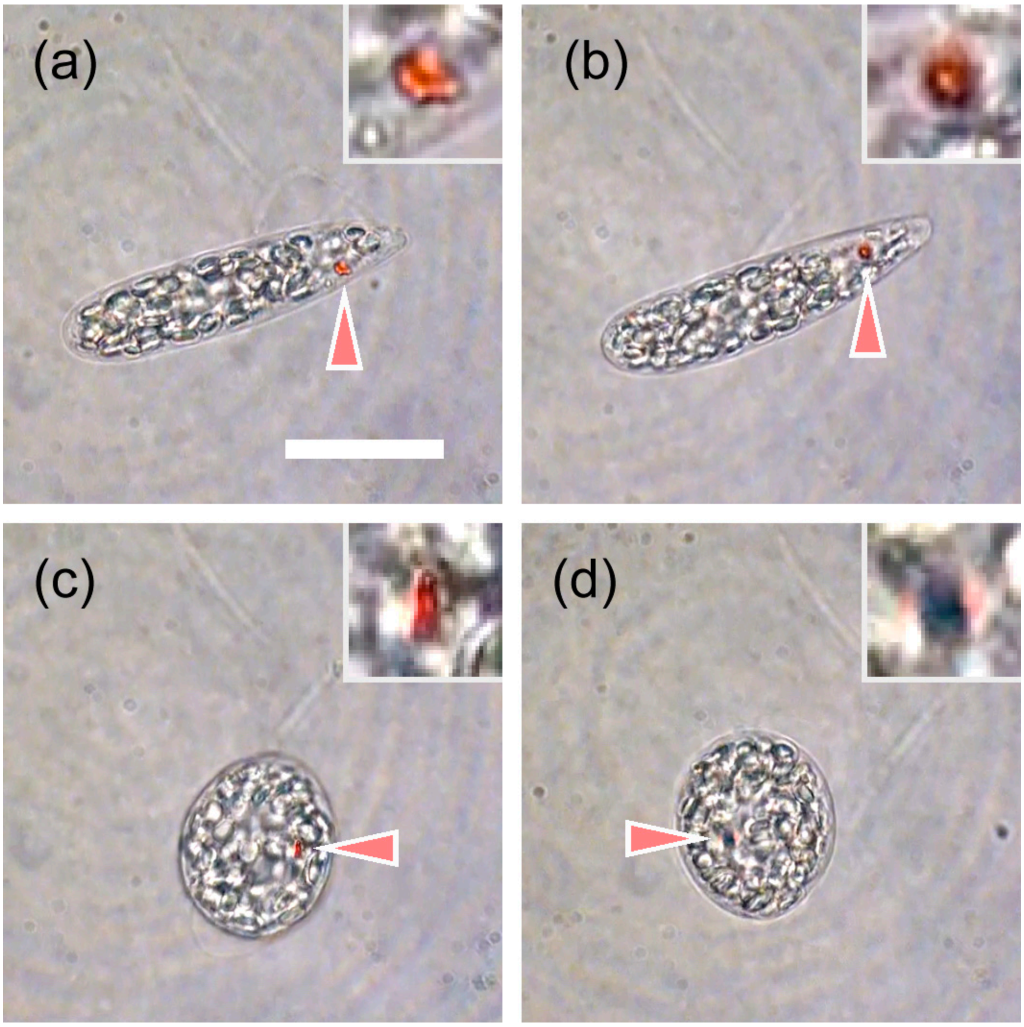

Figure S2. Variation of eyespot appearance for a non-mitotic SM-ZK cell; observed as a small red ball composite (a), dark red sphere (b), red rod (c), and dark gray sheet (d). Scale bar, 20 μm.

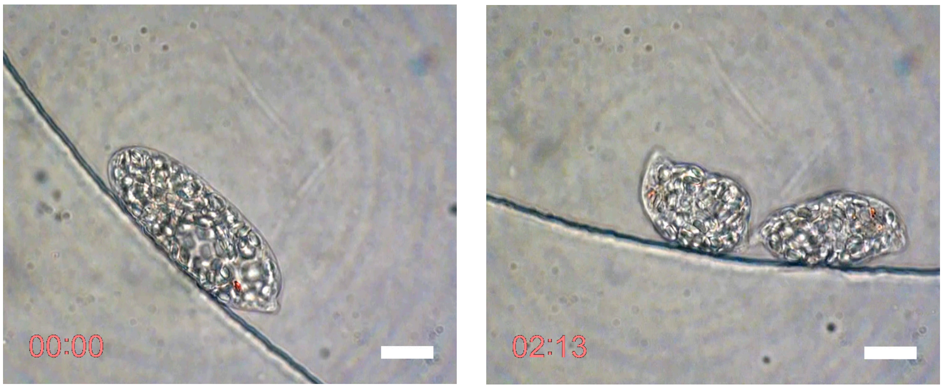

Movie S3, supplied by separate file Movie_66_S3.mov. Cell division observed for an SM-ZK cell in the microchamber. The movie was reproduced in a time-lapse manner from the original video-recording, with a 41 times higher playback speed. Slow frame advance playback is recommended. Time stamp in the movie represents hh:mm. Scale bar, 10 μm.

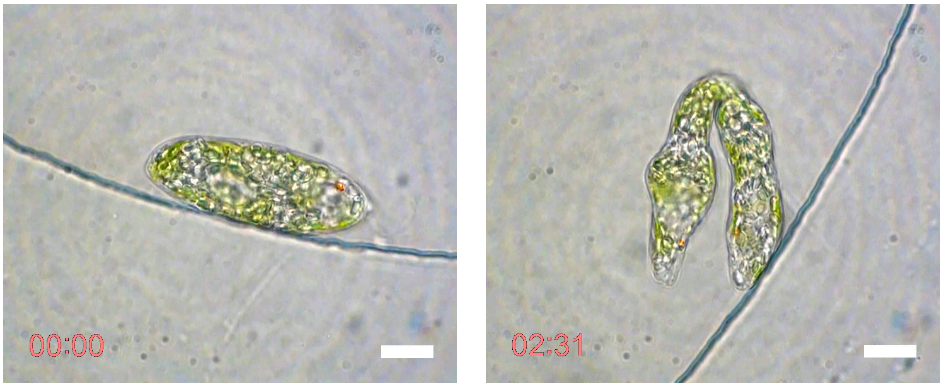

Movie S4, supplied by separate file Movie_66_S4.mov . Cell division observed for WT cell in the microchamber. The movie was reproduced in a time-lapse manner from the original video-recording, with a 41 times higher playback speed. Slow frame advance playback is recommended. Time stamp in the movie represents hh:mm. Scale bar, 10 μm.

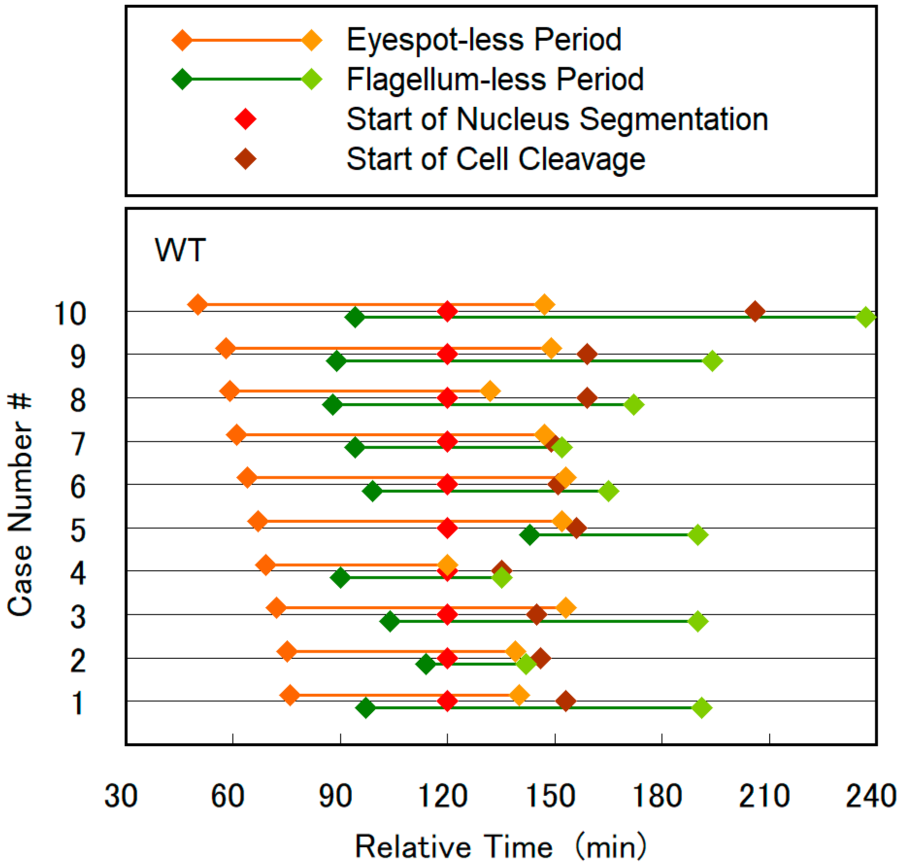

Figure S5. The period of disappearance of the eyespot and flagellum plotted for 10 WT cells, together with the initiation time of nucleus segmentation and cell cleavage. In the plot, the initiation time of nucleus segmentation was used as the standard timing (120 min). The eyespot disappeared 44–70 min (78 min in average, 15 min in deviation) before the nucleus segmentation for WT, and eyespot-less period remained for 51–97 min (48 min in average, 18 min in deviation). The case #4 corresponds to Figs. S1 and 7 and movie S4.

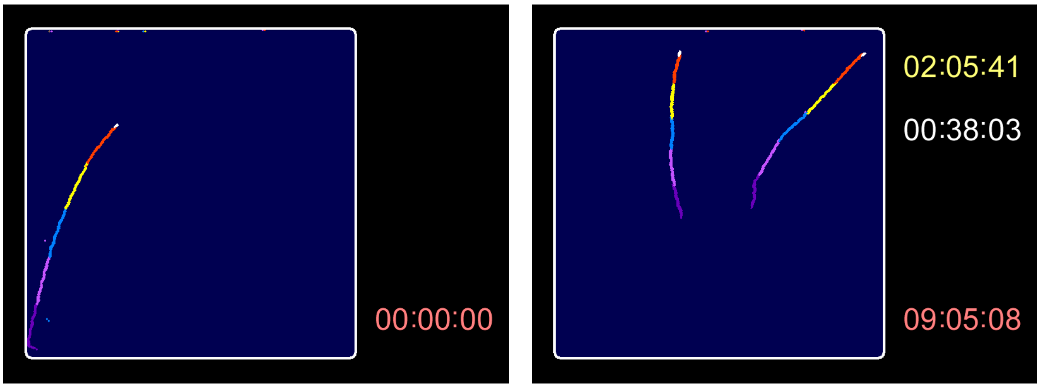

Movie S6, supplied by separate file Movie_66_S6.mov Swimming traces observed under a long-term millimeter-scale observation, including the period of cell division. The time index in the movie is provided in the format hh:mm:ss. The parental cell stopped swimming at 03:17:53, and cell division occurred at the location marked by a circle. The first daughter cell started swimming at 05:23:47, and thus the duration of cell division in this movie was 126 min. The second daughter cell started swimming at 06:01:50, and thus the difference in swimming start times between the first and second daughter cell was 38 min.

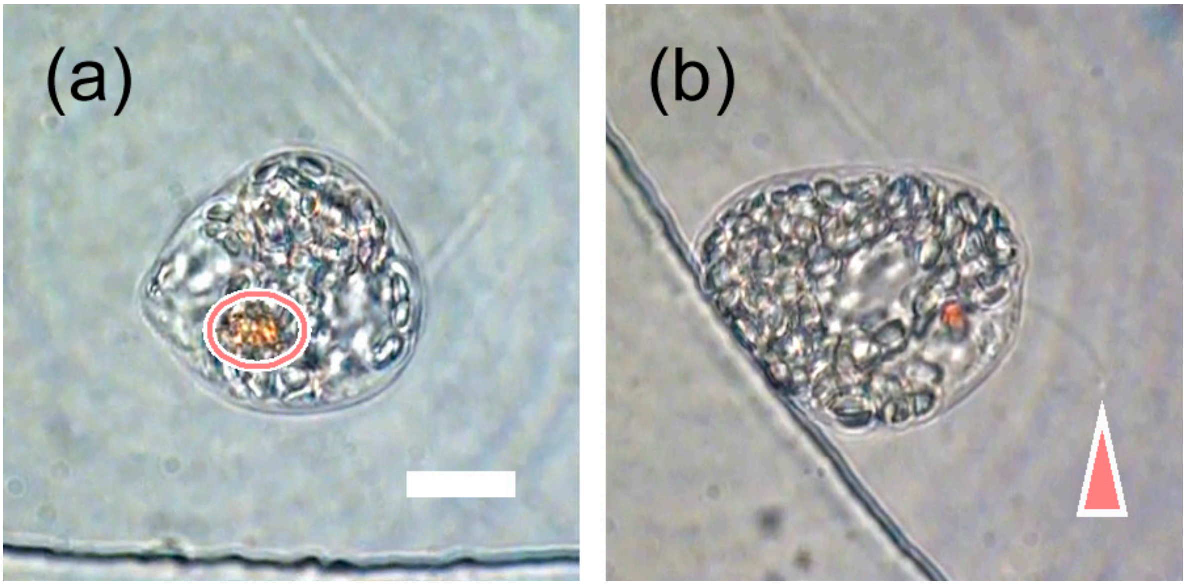

Figure S7. (a) Reddish brown hazy region (encircled) observed before the reappearance of a new distinctive eyespot (after the nucleus segmentation and before cell cleavage). (b) White dim spot (indicated by an arrow) observed at the outer end of shortened flagellum. Scale bar, 10 μm.
