# Supplementary figures and images for: Regeneration of the Eyespot and Flagellum in Euglena gracilis during Cell Division
Source: Plants (Basel). 2021 Sep 24;10(10):2004. doi: 10.3390/plants10102004 (PMC8537169; doi:10.3390/plants10102004)

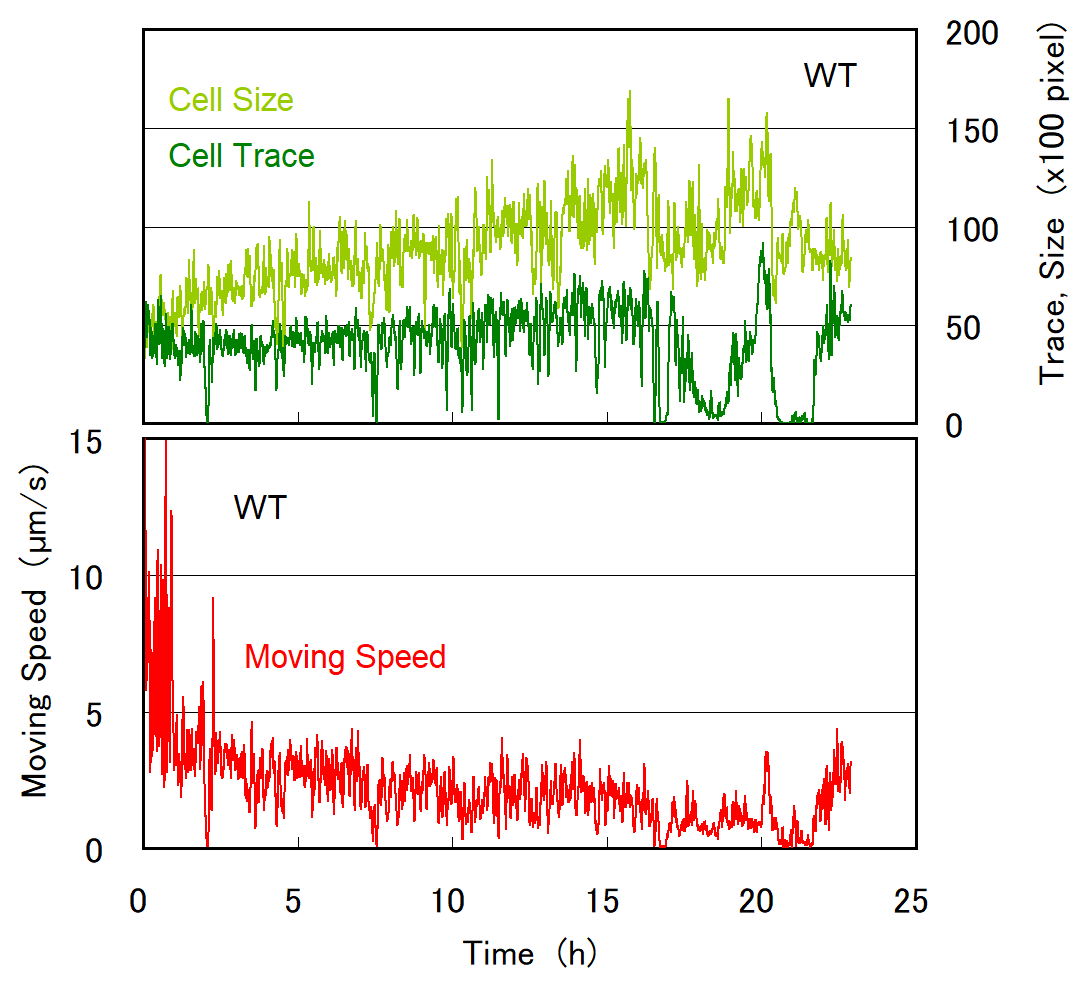

Supplement: Supplementary file 1 [file plants-10-02004-s001.zip › Fig_66_S1.png]

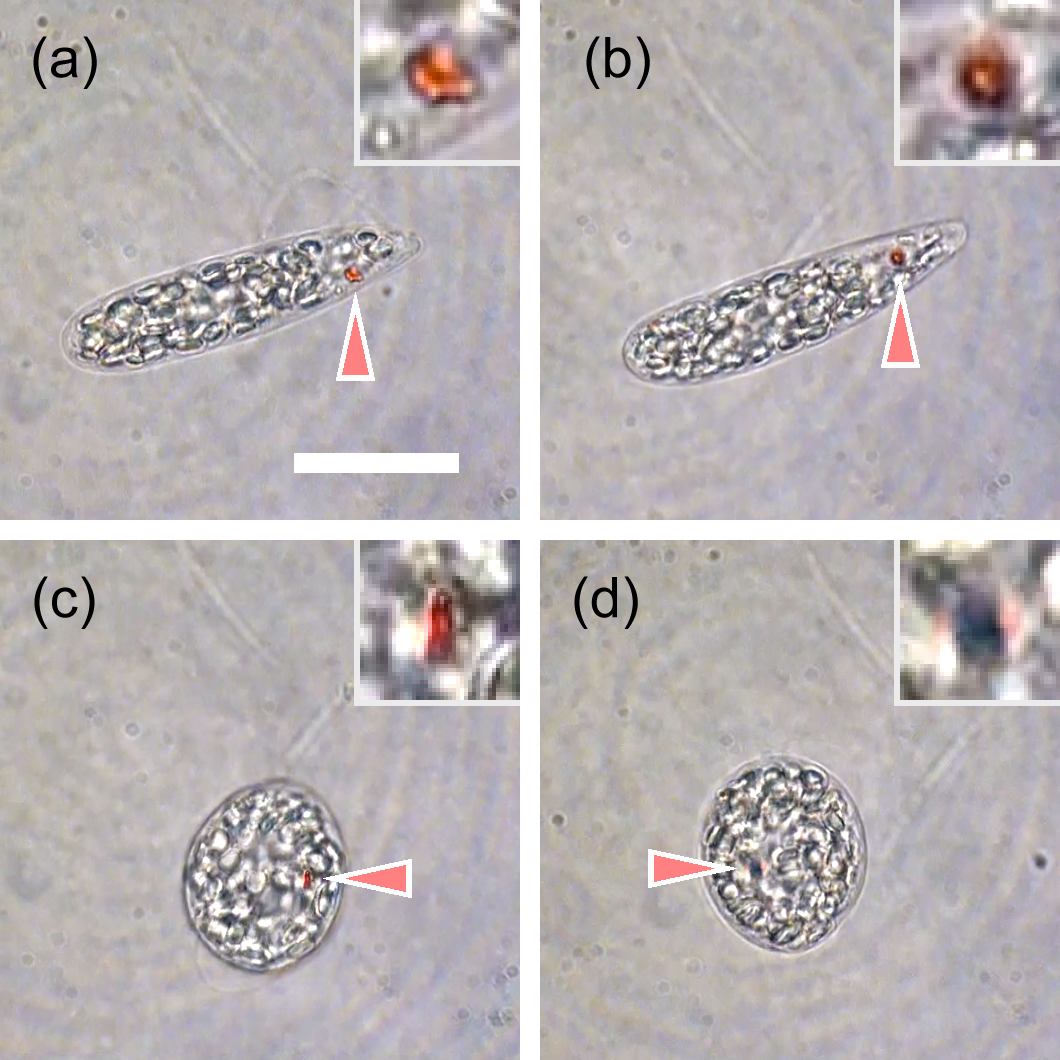

Supplement: Supplementary file 1 [file plants-10-02004-s001.zip › Fig_66_S2.png]

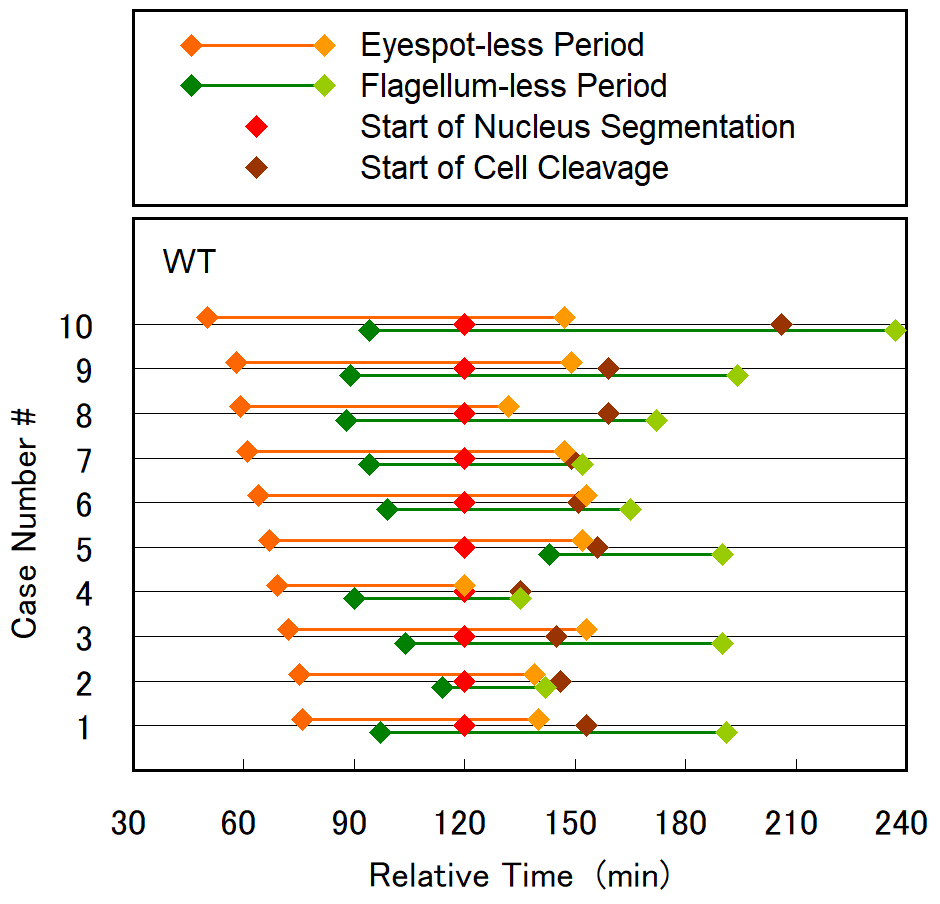

Supplement: Supplementary file 1 [file plants-10-02004-s001.zip › Fig_66_S5.png]

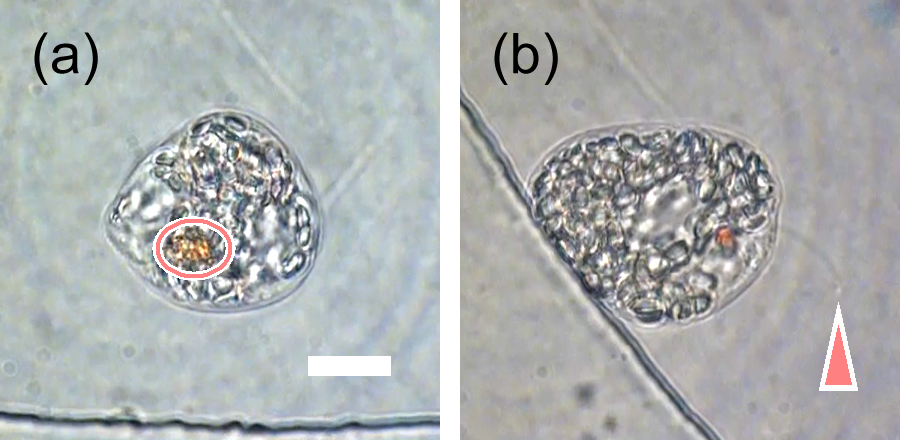

Supplement: Supplementary file 1 [file plants-10-02004-s001.zip › Fig_66_S7.png]
